# The PROtective VEntilation (PROVE) Network - advancing research and collaboration in mechanical ventilation

**DOI:** 10.62675/2965-2774.20250399

**Published:** 2025-08-18

**Authors:** Marcus J. Schultz, Lorenzo Ball, Martin Bernardi, Denise Battaglini, Laura A. Buiteman, Juliana Carvalho Ferreira, Marcelo Gama de Abreu, Silvia De Rosa, Sabrine N. Hemmes, Robert Huhle, Guido Mazzinari, David M.P. van Meenen, Prashant Nasa, Ary Serpa, Paolo Pelosi, Frederique Paulus, Chiara Robba, Patricia R. M. Rocco, Martin Scharffenberg, Edda Tschernko, Jakob Wittenstein

**Affiliations:** 1 Amsterdam UMC Locatie AMC Amsterdam Netherlands Amsterdam UMC Locatie AMC - Amsterdam, North Holland, Netherlands.; 2 Ospedale Policlinico San Martino Genova Italia Ospedale Policlinico San Martino - Genova, Liguria, Italia.; 3 Medical University of Vienna Vienna Austria Medical University of Vienna - Vienna, Austria.; 4 Reinier de Graaf Groep Delft Netherlands Reinier de Graaf Groep - Delft, South Holland, Netherlands.; 5 Universidade de São Paulo São Paulo SP Brazil Universidade de São Paulo - São Paulo (SP), Brazil.; 6 Cleveland Clinics Cleveland Ohio United States Cleveland Clinics - Cleveland, Ohio, United States.; 7 University of Trento Trento Italia University of Trento - Trento, Trentino Alto Adige, Italia.; 8 AVL Ziekenhuis Amsterdam Netherlands AVL Ziekenhuis - Amsterdam, North Holland, Netherlands.; 9 TUD Dresden University of Technology Dresden Germany TUD Dresden University of Technology - Dresden, Germany.; 10 Universidad de Valencia Valencia Spain Universidad de Valencia - Valencia, Comunidad Valenciana, Spain.; 11 The Royal Wolverhampton NHS Trust Wolverhampton United Kingdom The Royal Wolverhampton NHS Trust - Wolverhampton, United Kingdom.; 12 Monash University Melbourne Victoria Australia Monash University - Melbourne, Victoria, Australia.; 13 Universidade Federal do Rio de Janeiro Rio de Janeiro RJ Brazil Universidade Federal do Rio de Janeiro - Rio de Janeiro (RJ), Brazil.; 14 Universitätsklinikum Carl Gustav Carus Dresden Sachsen Germany Universitätsklinikum Carl Gustav Carus - Dresden, Sachsen, Germany.

**Keywords:** Respiration, artificial, Network, Research, Cooperative behavior, Artificial Intelligence, Innovation, Leadership, Mentors, Developing countries, Critical care, Operating rooms, Intensive care units

## Abstract

The PROtective VEntilation (PROVE) Network is a globally-recognized collaborative research group dedicated to advancing research, education, and collaboration in the field of mechanical ventilation. Established to address critical questions in intraoperative and intensive care ventilation, the network focuses on improving outcomes for patients undergoing mechanical ventilation in diverse settings, including operating rooms, intensive care units, burn units, and resource-limited environments in low- and middle-income countries. The PROVE Network is committed to generating high-quality evidence through a comprehensive portfolio of investigations, including randomized clinical trials, observational research, and meta-analyses. Its work has significantly contributed to understanding optimal ventilation strategies in critically ill patients, such as those with COVID-19, and in exploring innovative approaches like closed-loop ventilation systems. The network has spearheaded pioneering studies that have shaped clinical practice worldwide by integrating expertise from a wide range of disciplines. A defining feature of the PROVE Network is its emphasis on mentorship and collaboration. It fosters a supportive environment where junior researchers are guided by experienced mentors, ensuring the transfer of knowledge and promoting inclusivity. The network prioritizes gender balance and diversity, recognizing the value of varied perspectives in driving meaningful innovation and advancing research excellence. This paper reviews the history, key projects, and leadership of the PROVE Network, highlighting its impactful contributions to the field of mechanical ventilation. By uniting researchers globally, the PROVE Network exemplifies the power of collaboration in addressing complex clinical challenges, including personalized ventilation and the use of Artificial Intelligence, and improving patient care.

## INTRODUCTION

Mechanical ventilation (MV) is a cornerstone in the care of patients under general anesthesia for surgery, as well as for critically ill patients with respiratory failure related to acute respiratory distress syndrome (ARDS), sepsis, or other life-threatening illnesses. Over the years, significant strides have been made in improving ventilation strategies that can protect the lungs from ventilator-induced lung injury (VILI) while also meeting the patient's oxygenation and ventilation needs.^(1,2)^ However, challenges remain in optimizing ventilation practices across diverse patient populations, particularly in complex or high-risk cases, including those involving patients undergoing challenging surgeries, pediatric patients, burn victims, and individuals in low- and middle-income countries (LMICs).^(3)^ Therefore, research in MV is not just important – it is critical for improving patient outcomes and developing strategies that are applicable in a wide range of settings.

Mechanical ventilation plays a pivotal role in both the operating room and the intensive care unit (ICU), supporting patients through critical phases of care and recovery. In the operating room, it ensures proper ventilation during surgery, where factors such as one-lung ventilation or patient positioning can complicate management. In the ICU, ventilation strategies are key to managing patients with respiratory failure and mitigating complications such as VILI. These settings require continuous innovation to refine MV strategies, integrate novel technologies, and translate research findings into clinical practice. Given the complexity and diversity of clinical and scientific environments, collaboration is essential in advancing research in MV.

Successful research requires contributions from a diverse group of clinicians, researchers, and experts who offer various perspectives, methodologies, and experiences to the table.^(4)^ Senior researchers play a crucial role in mentoring the next generation of investigators, ensuring that knowledge and skills are passed on and that promising new researchers are nurtured. However, mentorship is most effective when there is a balance, particularly in terms of gender diversity. Research networks that prioritize inclusive mentoring practices, in which both junior and senior members collaborate and contribute, help foster innovation, inclusion, and diverse ideas.^(5–7)^ Despite the growing recognition of the importance of mentorship and balanced team dynamics, only some research networks have successfully integrated these values on such a large scale.

The PROtective VEntilation (PROVE) Network^(8)^ stands out as a collaborative effort that bridges the gap between established experts and emerging researchers while ensuring a balanced, inclusive, and diverse environment. As a global consortium, it offers an exceptional model of collaboration and mentorship, facilitating groundbreaking research in MV across various clinical contexts. This paper will explore the history, projects, and leadership of the PROVE Network, highlighting its contributions to the field of MV and its commitment to advancing research through collaboration, mentorship, and innovation.

### History of PROVE Network

The PROVE Network was established to address the growing need for collaborative research in MV, particularly in perioperative and intensive care settings. The network grew from recognizing that while individual research studies in ventilation are valuable, global collaboration can accelerate progress and lead to more impactful findings. Formed by leading experts in anesthesia, critical care and pulmonary medicine, the PROVE Network's primary mission is to enhance the understanding and clinical application of MV to improve patient outcomes across diverse patient populations.^(8)^

The origins of the PROVE Network can be traced to the collaborative efforts of five individuals who played a pivotal role in its founding. The late Prof. Paolo Pelosi, Prof. Marcelo Gama de Abreu, Prof. Marcus Schultz, Prof. Ary Serpa Neto, and Dr. Sabrine Hemmes initiated the network through their work on two landmark projects in 2012: the international multicenter randomized clinical trial PROVHILO and the global observational study LAS VEGAS.^(9,10)^ These foundational projects were supported by the European Society of Anaesthesiology and Intensive Care (ESAIC)^(11)^ through several grants, as well as by the former Academic Medical Center (AMC), now part of the Amsterdam University Medical Centers^(12)^ in the Netherlands. Notably, the financial model behind the network activities also reflects its collaborative nature, with partners from around the world joining the projects using local grants and departmental funds. Over time, the Network's interest expanded from perioperative care to intensive care settings, with notable contributions to projects such as the international randomized clinical trials NEBULAE,^(13,14)^ PReVENT,^(15,16)^ and RELAx,^(17,18)^ and global observational studies like PRoVENT^(19)^ and PRoVENT-iMiC^(20–22)^ (Table 1).

**Table 1:** Selection of completed and running studies and projects ran by the PROVE Network-investigators

Study	Design	Location, intervention	N	Current status					
				Registration	Status	Primary analysis	Secondary analyses	Post hoc analyses	Harmonized & merged
PROVHILO	International RCT	OR, PEEP	900	NCT01441791	Completed	✓	✓	Open	REPEAT
PROBESE	International RCT	OR, PEEP	1976	NCT02148692	Completed	✓	✓	Open	REPEAT
PROVAR	Single center RCT	OR, variable ventilation	50	NCT01683578	Completed	✓	-	-	-
PROTHOR	International RCT	OR, PEEP	2378	NCT02963025	Completed	Pending	Pending	Pending	REPEAT
LoCo	Single center RCT	OR, V_T_, and PEEP	30	NTR 4391	Completed	✓	✓	-	-
DESIGNATION	International RCT	OR, PEEP	1468	NCT03884543	Running	Pending	Pending	Pending	REPEAT
GENERATOR	International RCT	OR, PEEP	1806	NCT06101511	Running	Pending	Pending	Pending	REPEAT
PROFLOW-robotic	International RCT	OR, flow-controlled ventilation	TBD	NCT06703814	Pending	-	-	-	REPEAT
NEBULAE	National RCT	ICU, nebulization	922	NCT02159196	Completed	✓	-	Open	wizARDS
PReVENT	National RCT	ICU, V_T_	961	NCT02153294	Completed	✓	✓	Open	wizARDS
RELAx	National RCT	ICU, PEEP	980	NCT03167580	Completed	✓	✓	Open	wizARDS
ICONIC	International RCT	ICU, restricted oxygen use	646	NTR7376	Completed	✓	✓	Open	-
POSITiVE	Single center RCT	ICU, closed-loop ventilation	220	NCT03180203	Completed	✓	✓	-	POSITiVEs
INTELLiSTREAM	Single center RCT	ICU, closed-loop ventilation	73	NCT04599491	Completed	✓	✓	Open	-
INTELLiPOWER	National RCT	ICU, closed-loop ventilation	96	NCT04827927	Completed	✓	✓	Open	-
ACTiVE	International RCT	ICU, closed-loop ventilation	1200	NCT04593810	Running	Pending	Pending	Pending	-
HEPBURN	International RCT	Burn ICU, nebulization	13	NCT01773083	Completed	✓	-	-	-
PERMISS	International RCT	ICU, RR	TBD	To follow	Planned	Pending	Pending	Pending	-
POSITiVE II	International RCT	ICU, closed-loop ventilation	328	NCT06178510	Running	Pending	Pending	Pending	POSITiVEs
VIPS	International RCT	ICU, variable ventilation	228	NCT01769053	Completed	Pending	Pending	Pending	-
PReSPON	International RCT	ICU, spontaneous ventilation	840	NCT06027008	Running	Pending	Pending	Pending	-
LAS VEGAS	International observational	OR	10520	NCT01601223	Completed	✓	✓	Open	LapRas
AVATaR	International observational	OR	1015	NCT02989415	Completed	✓	✓	Open	LapRas
BIG APPLE	International observational	Pediatric OR	TBD	To follow	Running	Pending	Pending	Pending	-
AIR TEST	Single center observational	OR	623	NCT05345743	Completed	Pending	-	Pending	-
PRoVENT	International observational	ICU	935	NCT01868321	Completed	✓	✓	Open	PRoPERLy I and II & PRIME
PRoVENT-iMiC	International observational	ICU	1315	NCT03188770	Completed	✓	✓	Open	PRoPERLy I and II & PRIME
LAMINAR	International observational	Burn ICU	160	NCT02312869	Completed	✓	-	-	-
ENIO	International observational	ICU	1512	NCT03400904	Completed	✓	✓	Open	PRIME
VESPer	International observational	Pediatric ICU	166	-	Completed	✓	-	-	-
PRoVENT-PED	International observational	Pediatric ICU	TBD	NCT06220825	Running	Pending	Pending	Pending	-
REPOrt	International observational	ICU	TBD	To follow	Planned	Pending	Pending	Pending	-
PRoVENT 2+	International observational	ICU	TBD	To follow	Planned	Pending	Pending	Pending	-
PRoVENT-COVID	National observational	ICU	1022	NCT04346342	Completed	✓	✓	Open	PRoVAcT
PRoAcT-COVID	National observational	ICU	946	NCT04719182	Completed	✓	✓	Open	PRoVAcT
BRAVE	Single center crossover	ICU, closed-loop ventilation	20	NCT06367816	Completed	Pending	Pending	Pending	-
AIRDROP	Single center crossover	ICU, closed-loop ventilation	13	NCT03211494	Completed	✓	-	Open	-
DIOS	Delphi	ARDS definitions	N.A.	NCT06159465	Completed	✓	N.A.	N.A.	N.A.
ACCordingLy	Delphi	Airway care in ICU patients	N.A.	NCT06649734	Running	Pending	N.A.	N.A.	N.A.
AEScuLApius	Delphi	Ventilator settings in ICU patients	N.A.	To follow	Planned	-	N.A.	N.A.	N.A.
PrECiSIOn	Delphi	PPC definitions	N.A.	To follow	Planned	-	N.A.	N.A.	N.A.
PRORVNet	Delphi	RV failure in ECMO	N.A.	NCT05948332	Completed	✓	N.A.	N.A.	N.A.

For the original studies and the full name of each study or project, follow the NCT (https://clinicaltrials.gov) or NTR (https://www.onderzoekmetmensen.nl/en) number.

‘✓’ means that the analysis is published, go to the website of PROVE Network (www.provenetwork.org) to find the peer-reviewed publication; ‘pending’ means that the study or project is currently conducted or that the primary analysis is awaited or not yet published, or that secondary analysis or *post hoc* analyses are not yet performed or not yet possible; ‘N.A.’ means that the option is not applicable; ‘TBD’ means that the sample size still needs to be established.

OR - operating room; PEEP - positive end-expiratory pressure; V_T_ - tidal volume; ICU - intensive care unit; RR - respiratory rate FCV - flow-controlled ventilation; TBD - to be determined; ARDS - acute respiratory distress syndrome; PPC - postoperative pulmonary complications; RV - right ventricle; ECMO - extracorporeal membrane oxygenation.

The PROVE Network has also maintained a strong interest in preclinical research, which has been a cornerstone of its scientific contributions. Several investigators involved in the network's initiation have advanced this focus, collaborating with leading laboratories worldwide to deepen the understanding of MV at a fundamental level. Notable partnerships include the Laboratory of Experimental Intensive Care and Anesthesiology (L·E·I·C·A) in Amsterdam,^(23)^ the Pulmonary Engineering Group (PEG) in Dresden,^(24)^ the laboratory of Patricia Rocco at the *Universidade Federal do Rio de Janeiro*, Brazil, and the laboratory of Edda Tschernko in Vienna.^(25)^ These collaborations have facilitated groundbreaking work in experimental models,^(26–31)^ providing critical insights into the mechanisms of VILI and the optimization of ventilation strategies, thereby complementing the network's clinical research endeavors.

**Figure 1:**
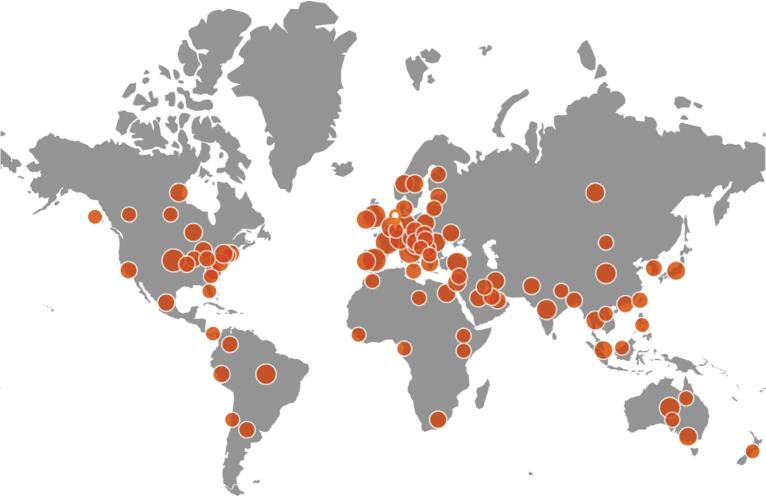
PROVE Network - a global network of research centers across six continents, actively engaged in groundbreaking clinical investigations and innovative preclinical studies.

The network's foundation has always been built on the principles of collaboration, mentorship, and inclusivity, with a special emphasis on supporting the next generation of researchers. By fostering an environment where senior researchers actively mentor junior members, the PROVE Network ensures the sustainability and growth of the field. This collaborative model, which combines expertise from around the world (Figure 1), has yielded a growing body of research that has significantly shaped the field of MV.

### Key projects and research areas

The PROVE Network has initiated, led, and continues to lead a wide range of clinical and preclinical studies aimed at improving MV strategies across various clinical settings. The network's projects include randomized clinical trials, observational studies, meta-analyses and analyses of merged databases that provide a comprehensive view of ventilation practices and outcomes. Recently, the network-initiated Delphi-based expert consensus studies and collaborated on cross-sectional surveys. The network also emphasizes personalized medicine approaches, the development of protocols tailored for LMICs, and the resolution of ethical challenges in global clinical trials. Its efforts aim to promote equitable access to research participation and ensure that the benefits of advancements in care are fairly distributed across all populations. Last but not least, the strong collaboration between the several laboratories leads to numerous successful projects.

#### Randomized clinical trials and observational studies

The PROVE Network has played a pivotal role in conducting studies to evaluate the effectiveness of various ventilation strategies in improving patient outcomes. These studies are designed to assess various interventions, including lung-protective ventilation strategies, the use of closed-loop systems, the impact of personalized ventilation on patient recovery, and the application of Artificial Intelligence in adjusting the ventilator. Parallel to these randomized clinical trials, observational studies are also conducted to gather objective-world evidence on ventilation practices and their impact on patient survival and recovery. Dr. Chiara Robba and Dr. Denise Battaglini lead the randomized clinical trial- section, guiding the development and implementation of these high-impact studies. Dr. Sabrine Hemmes and Dr. Lorenzo Ball oversee the observational studies, ensuring a rigorous analysis of ventilation practices across various settings and patient populations.

The PROVE Network is currently conducting multiple randomized clinical trials in the operating room and the intensive care unit, exploring various ventilator settings and novel ventilation modes.

#### Meta-analyses and merged databases

The PROVE Network's meta-analyses and merged databases have significantly contributed to the understanding of ventilation strategies in critical care. Under the leadership of Prof. Ary Serpa Neto, Dr. Guido Mazzinari, and Dr. David van Meenen, the network has conducted extensive-scale analyses that combine data from multiple studies to provide more robust conclusions on the effectiveness of various ventilation techniques. These meta-analyses help to identify the best practices and inform evidence-based guidelines that can be used globally.

#### Special areas of focus

In addition to general MV research, the PROVE Network has focused on several specialized areas, including:

– Closed-loop ventilation: PROVE Network is actively engaged in research on closed-loop ventilation, exploring its potential to optimize respiratory support in critically ill patients. Dr. Laura Buiteman has taken the lead in these efforts, driving forward studies that aim to refine and advance automated ventilation strategies, improve patient outcomes, and enhance clinical practice.
– Ventilation in coronavirus disease 2019 (COVID-19): PROVE network has conducted several studies examining the optimal ventilation strategies for patients with COVID-19, particularly in the ICU setting. Prof. Frederique Paulus has led the efforts in this area, ensuring that findings are integrated into global response strategies.
– Ventilation in burn patients: burn patients present unique challenges in ventilation, and the network has conducted studies to explore strategies that minimize VILI in these critically ill individuals.
– Ventilation in children: the PROVE Network has explored pediatric ventilation strategies, focusing on optimizing care for critically ill children requiring MV. Dr. David van Meenen heads this section in close collaboration with pediatric intensivists across the globe.
– Ventilation in neurocritical care: patients in neurocritical care, particularly those with brain injuries, present unique challenges in ventilation. Led by Dr. Chiara Robba, the network has conducted studies to explore strategies that optimize ventilation and minimize ventilator-associated complications in these critically ill individuals, focusing on protecting brain function and supporting recovery.
– Sex inequities: PROVE Network is dedicated to investigating sex-based differences in ventilatory support, seeking to understand how these inequities impact outcomes in critically ill patients. Studies in this area aim to identify disparities in treatment approaches and outcomes, providing evidence to guide more equitable and effective respiratory care practices.
– Ventilation in resource-limited settings: in LMICs, where resources may be scarce, the network has worked to develop ventilation strategies that are both effective and feasible in settings with low resources.
– Alarms and alarm management: PROVE Network is committed to advancing research on alarms and alarm management, recognizing the critical importance of creating a ‘silent’ ICU to enhance patient comfort, reduce alarm fatigue, and improve sound hygiene. Laura Buiteman is leading efforts in this area, focusing on innovative strategies to optimize alarm systems and minimize unnecessary noise in the ICU environment, addressing an issue of growing relevance in critical care.
– Patient-ventilator asynchrony: PROVE Network is actively investigating patient-ventilator asynchrony, with Prof. Juliana Ferreira leading efforts to understand its impact on patient outcomes. This research aims to uncover the clinical significance of asynchronies and develop strategies to optimize synchrony between patients and ventilators, ultimately improving care for critically ill patients.
– Physiotherapy and rehabilitation in invasively ventilated patients: invasive ventilation requires specialized physiotherapy and rehabilitation strategies to support recovery and prevent complications. Led by Dr. Denise Battaglini, the network conducts studies to investigate effective rehabilitation techniques aimed at improving functional outcomes and accelerating recovery in critically ill patients undergoing MV.^(32)^
– Led by Dr. Silvia De Rosa and Dr Martin Bernardi, the network is expanding its focus on the intricate relationship between the lungs and kidneys in critically ill patients. Future studies aim to investigate the effects of lung-protective ventilation strategies on renal perfusion and function, with a particular emphasis on patients with acute kidney injury requiring renal replacement therapies. These investigations will explore kidney lung crosstalk, the timing and personalization of renal replacement therapies to optimize outcomes, and the impact of fluid management strategies on improving respiratory mechanics. Additionally, the association between fluid balance,^(33,34)^ creep fluid,^(35)^ and outcomes in ventilated patients will be a key area of interest, aiming to better understand how fluid accumulation influences respiratory function and recovery.

### Delphi studies

PROVE Network conducts Delphi projects to build consensus among international experts on important perioperative and intensive care medicine topics. These projects define essential clinical outcomes, identify research priorities, and establish standardized protocols or guidelines. The results of these collaborative efforts aim to guide future studies and improve clinical practice by addressing areas of uncertainty and variability in care. Dr. Prashant Nasa, a Delphi-expert, leads this section.

### Surveys

PROVE Network is actively involved in designing and conducting surveys to gather critical insights on various topics in critical care. These surveys, led by Dr. Denise Battaglini and Dr. Silvia de Rosa, aim to collect data from clinicians, researchers, and other stakeholders to understand better current practices and challenges in areas such as ventilation strategies, patient care, and treatment protocols. By leveraging a broad network of professionals, the surveys help identify knowledge gaps and inform the development of evidence-based solutions relevant to both clinical and research settings. This collaborative approach ensures that the findings are robust and widely applicable, providing valuable insights to shape future studies and guidelines in anesthesia and critical care.

### Preclinical studies and Artificial Intelligence

Preclinical studies are crucial in advancing medical research, particularly in MV. These studies help bridge the gap between laboratory research and clinical practice by testing interventions in controlled environments before they are applied in human trials. PROVE Network focuses on several preclinical investigations, including those related to ventilation strategies and automated ventilation systems. A key aspect of these studies is the evaluation of new technologies designed to optimize care in patients who are invasively ventilated. Key players in this context include Prof. Marcelo Gama de Abreu, Prof. Patricia Rocco, Dr. Martin Scharffenberg, Prof. Edda Tschernko, and Dr. Jakob Wittenstein.

Next, the network evaluates the Artificial Intelligence algorithm, which aims to refine ventilator settings for mechanically ventilated patients by leveraging data from retrospective studies across diverse populations. Through these efforts, the PROVE Network enhances the safety, efficiency, and personalization of MV, setting the foundation for future clinical applications. These Intellilung projects, funded by the European Union, are led by Prof. Marcelo Gama de Abreu, Dr. Robert Huhle, Dr. Martin Scharffenberg and Dr. Jakob Wittenstein.

### Thematic leadership and mentorship

A core strength of the PROVE Network is its dedication to mentorship and fostering a diverse research environment. Senior researchers guide and mentor junior members, ensuring a steady flow of knowledge while empowering the next generation of researchers. Gender balance is prioritized within the network, creating an inclusive environment where both men and women can contribute to and lead research initiatives.

## DISCUSSION

**Figure 2:**
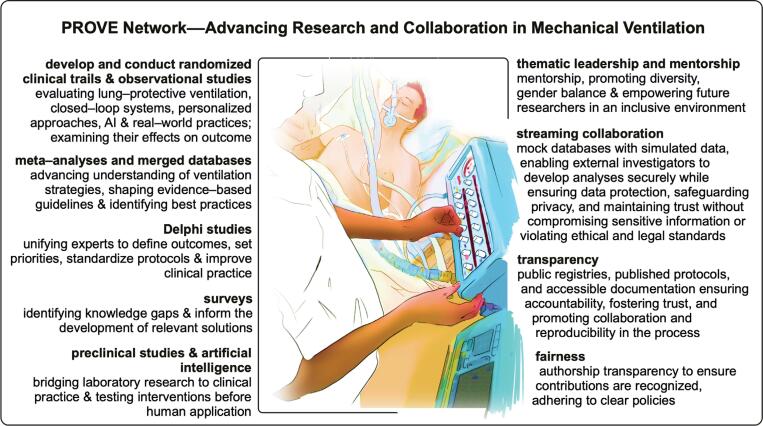
The PROVE Network's approach to advancing research and collaboration in mechanical ventilation.

The PROVE Network represents a unique model for advancing MV research through collaboration, mentorship, and a strong focus on inclusivity and diversity (Figure 2). Its interdisciplinary approach allows researchers to tackle complex questions across a variety of clinical contexts, ultimately advancing the science and clinical practice of MV. Through its focus on both high-quality studies and real-world observational data, the network is paving the way for more personalized and effective ventilation strategies, addressing gaps in care in both high-resource and low-resource settings.

The vast number of merged databases within the PROVE Network offers valuable opportunities for secondary analyses, but this also comes with significant responsibilities. Requests for secondary analyses from investigators outside the network require careful consideration, particularly regarding data sharing. More than simply granting access to the data is often not the optimal approach, as other teams have collected portions of the data, and there are significant privacy considerations. Ensuring compliance with data protection regulations and safeguarding patient confidentiality is paramount, requiring strict oversight of how the data is accessed and used. Sharing sensitive information without appropriate safeguards could lead to breaches of privacy, raising ethical and legal concerns that could jeopardize trust and collaboration within the network.

Additionally, navigating the associated bureaucratic challenges - such as establishing sharing agreements and addressing concerns from privacy officers and legal departments - can lead to substantial delays. A practical solution involves using a synthetic data generation process. Synthetic data replaces some or all observed values by sampling from probability distributions that retain the essential statistical properties of the original data while ensuring that no sensitive information is disclosed to third parties. These "mock databases", filled with simulated data, enable external investigators to develop analysis scripts without needing to access the original datasets. The PROVE Network can then run these scripts on real data and share the results, ensuring both efficient collaboration and data protection.

Transparency is a cornerstone of all projects within the PROVE Network, ensuring accountability and fostering trust in the research process. Each project is registered on a public registry, clearly outlining the hypotheses to be tested, the outcomes being measured, and the number of patients required. To further enhance transparency, study protocols and analysis plans are typically submitted to peer-reviewed journals, making them accessible for scrutiny and reference. For ongoing projects, dedicated websites often serve as centralized hubs, providing access to critical documentation that can be downloaded and used locally. These practices collectively ensure that the research is conducted with openness and integrity while also facilitating collaboration and reproducibility.

The PROVE Network is committed to fairness and transparency in the assignment of authorships, adhering to clear policies that recognize the contributions of all involved while maintaining scientific integrity. Every project is expected to result in at least one scientific report detailing the main findings, strictly following the original analysis plan. Any additional analyses not outlined in the original plan are included as post hoc analyses within the primary report. The first and typically foremost report from a PROVE Network-initiated project is authored by a Writing Committee, generally a subgroup of the Steering Committee. In alignment with the PROVE Network's policy, the primary manuscript reporting on a randomized clinical trial or observational study is often published as a collaborative paper under the byline of the PROVE Network investigators without listing individual researchers. The Principal Investigator is identified as the contact person, and the members of the Steering Committee, the Writing Committee, and all local investigators from participating centers are acknowledged at the end of the manuscript or in an appendix, depending on the journal's policy. If a journal cannot accommodate this approach, alternative options are discussed with the Steering Committee, and a full explanation is provided on the project's website. For secondary manuscripts, such as those reporting on subgroup or secondary analyses, a different authorship structure may be used. These manuscripts can list individual authors but always include the phrase "on behalf of the PROVE Network investigators" to recognize the collaborative effort. All practices follow the Guidelines of the International Committee of Medical Journal Editors^(36)^ ensuring fairness and proper attribution of contributions.

The PROVE Network warmly invites researchers to join its collaborative community by registering. This opportunity connects investigators with a vibrant network focused on advancing research in perioperative MV and ventilation practices in the ICU. By joining, researchers gain access to upcoming initiatives, fostering multidisciplinary collaboration and contributing to impactful studies that aim to improve patient care in perioperative and critical care settings. Registration through the website is a step toward being part of a global effort to shape the future of MV research and innovation.

As the field of MV continues to evolve, the PROVE Network's dedication to mentorship, innovation, and collaboration will remain essential in driving future advancements. Its commitment to diverse leadership ensures that the network will continue to be at the forefront of research in MV, improving patient outcomes worldwide. Thus, whenever a given study is completed and published, the PROVE Network will not rest but prepare the next one. As we say in those moments: "to the next!".
